# Supplementary material for: Widespread transcript shortening through alternative polyadenylation in secretory cell differentiation
Source: Nat Commun. 2020 Jun 23;11:3182. doi: 10.1038/s41467-020-16959-2 (PMC7311474; doi:10.1038/s41467-020-16959-2)
Supplement: Supplementary file 1 — Supplementary Information [file 41467_2020_16959_MOESM1_ESM.pdf]

Supplementary Information

Widespread transcript shortening through alternative polyadenylation in secretory cell differentiation

Cheng *et al*.

Supplementary Tables

Supplementary Table 1. TB subtype marker genes

Subtype	Gene Symbol	Gene Name
EVT	*AIF1L*	allograft inflammatory factor 1 like
EVT	*ASCL2*	achaete-scute family bHLH transcription factor 2
EVT	*C12orf75*	chromosome 12 open reading frame 75
EVT	*CD9*	CD9 molecule
EVT	*CLDN19*	claudin 19
EVT	*DIO2*	iodothyronine deiodinase 2
EVT	*FN1*	fibronectin 1
EVT	*HAPLN3*	hyaluronan and proteoglycan link protein 3
EVT	*HLA-G*	major histocompatibility complex, class I, G
EVT	*HPGD*	15-hydroxyprostaglandin dehydrogenase
EVT	*ITGA1*	integrin subunit alpha 1
EVT	*ITGA5*	integrin subunit alpha 5
EVT	*JPT1*	Jupiter microtubule associated homolog 1
EVT	*LAIR2*	leukocyte associated immunoglobulin like receptor 2
EVT	*LVRN*	laeverin
EVT	*MMP2*	matrix metallopeptidase 2
EVT	*MT1E*	metallothionein 1E
EVT	*MYCNUT*	MYCN upstream transcript (non-protein coding)
EVT	*NOTUM*	notum, palmitoleoyl-protein carboxylesterase
EVT	*PTPRF*	protein tyrosine phosphatase, receptor type F
EVT	*PYCR1*	pyrroline-5-carboxylate reductase 1
EVT	*SLCO4A1*	solute carrier organic anion transporter family member 4A1
EVT	*TPM1*	tropomyosin 1
SCT	*CGA*	glycoprotein hormones, alpha polypeptide
SCT	*CGB5*	chorionic gonadotropin beta subunit 5
SCT	*CSH2*	chorionic somatomammotropin hormone 2
SCT	*CSHL1*	chorionic somatomammotropin hormone like 1
SCT	*CYP11A1*	cytochrome P450 family 11 subfamily A member 1
SCT	*CYP19A1*	cytochrome P450 family 19 subfamily A member 1
SCT	*DEPDC1B*	DEP domain containing 1B
SCT	*ERVW-1*	endogenous retrovirus group W member 1, envelope
SCT	*GDF15*	growth differentiation factor 15
SCT	*GH2*	growth hormone 2
SCT	*HSD3B1*	hydroxy-delta-5-steroid dehydrogenase, 3 beta- and steroid delta-isomerase 1
SCT	*KISS1*	KiSS-1 metastasis-suppressor
SCT	*KMO*	kynurenine 3-monooxygenase
SCT	*LGALS13*	galectin 13
SCT	*LGALS14*	galectin 14
SCT	*PSG1*	pregnancy specific beta-1-glycoprotein 1
SCT	*PSG11*	pregnancy specific beta-1-glycoprotein 11
SCT	*PSG3*	pregnancy specific beta-1-glycoprotein 3
SCT	*PSG4*	pregnancy specific beta-1-glycoprotein 4

SCT	*PSG6*	pregnancy specific beta-1-glycoprotein 6
SCT	*PSG9*	pregnancy specific beta-1-glycoprotein 9
SCT	*SDC1*	syndecan 1
SCT	*SERPINB2*	serpin family B member 2
VCT	*BCAM*	basal cell adhesion molecule (Lutheran blood group)
VCT	*BEX3*	brain expressed X-linked 3
VCT	*CTNNB1*	catenin beta 1
VCT	*DUSP9*	dual specificity phosphatase 9
VCT	*ELF5*	E74 like ETS transcription factor 5
VCT	*FGFR2*	fibroblast growth factor receptor 2
VCT	*FOXO4*	forkhead box O4
VCT	*FZD5*	frizzled class receptor 5
VCT	*GGCT*	gamma-glutamylcyclotransferase
VCT	*HMGA1*	high mobility group AT-hook 1
VCT	*ISYNA1*	inositol-3-phosphate synthase 1
VCT	*ITGA6*	integrin subunit alpha 6
VCT	*LRP5*	LDL receptor related protein 5
VCT	*MIR205HG*	MIR205 host gene
VCT	*PAGE4*	PAGE family member 4
VCT	*PARP1*	poly(ADP-ribose) polymerase 1
VCT	*PEG10*	paternally expressed 10
VCT	*SLC22A11*	solute carrier family 22 member 11
VCT	*SMAGP*	small cell adhesion glycoprotein
VCT	*TEAD4*	TEA domain transcription factor 4
VCT	*TINAGL1*	tubulointerstitial nephritis antigen like 1
VCT	*TP63*	tumor protein p63
VCT	*VGLL1*	vestigial like family member 1

Supplementary Table 2. Primers used in this study.

Gene Symbol	Targeting Region	Direction	Sequence (5’ -> 3’)
CGB	Coding	Forward	CGTCAACACCACCATCTGTG
CGB	Coding	Reverse	ATGGACTCGAAGCGCACAT
DNAJC3	Common	Forward	AGAAGTTTGACGACGGAGAAG
DNAJC3	Common	Reverse	GCCATGAGTTCCAGCTTCTGT
DNAJC3	aUTR	Forward	CCGAGCAAGGAGGAGGTAAA
DNAJC3	aUTR	Reverse	ACACGGGCATAAAAATAGTACCCA
ERVFRD-1	Coding	Forward	CCTTCACTAGCAGCCTACCG
ERVFRD-1	Coding	Reverse	GCTGTCCCTGGTGTTTCAGT
PLEKHA6	Common	Forward	CCTGCCACTTTGTCCGTCTC
PLEKHA6	Common	Reverse	TGGCAGAGGCGCAGACATTA
PLEKHA6	aUTR	Forward	ATTGGGGCCAATTTGATTTGCT
PLEKHA6	aUTR	Reverse	TGCTACAGCTGTTTGTCCCC
SPCS3	Common	Forward	TGCTCTGAACCAAGTTGTCCT
SPCS3	Common	Reverse	CCAGCATTTGGTACGACGTT
SPCS3	aUTR	Forward	TATGCTTTTTACGTGAAGCTGCTAA
SPCS3	aUTR	Reverse	AGTGTAACTAACCTCCCCCA
TIMP2	Common	Forward	CCATGATCCCGTGCTACATCT
TIMP2	Common	Reverse	GTCGAGAAACTCCTGCTTGG
TIMP2	aUTR	Forward	CTAAGCACAGCTCTCTTCTCCT
TIMP2	aUTR	Reverse	CAGCATAAACACAGTGCTCCC

Supplementary Table 3. USPS genes

Gene Symbol	Gene Name
ACSL3	acyl-CoA synthetase long chain family member 3
AGFG1	ArfGAP with FG repeats 1
AKAP9	A-kinase anchoring protein 9
AP1G1	adaptor related protein complex 1 subunit gamma 1
AP2A1	adaptor related protein complex 2 subunit alpha 1
ARF1	ADP ribosylation factor 1
ARF4	ADP ribosylation factor 4
ARFGEF2	ADP ribosylation factor guanine nucleotide exchange factor 2
ARL1	ADP ribosylation factor like GTPase 1
ATF6	activating transcription factor 6
ATP6V0D1	ATPase H+ transporting V0 subunit d1
BAIAP2	BAI1 associated protein 2
BCL2	BCL2 apoptosis regulator
BET1L	Bet1 golgi vesicular membrane trafficking protein like
BHLHA15	basic helix-loop-helix family member a15
BICD2	BICD cargo adaptor 2
BIN2	bridging integrator 2
CALR	calreticulin
CAMSAP2	calmodulin regulated spectrin associated protein family member 2
CD55	CD55 molecule (Cromer blood group)
CD59	CD59 molecule (CD59 blood group)
CEBPB	CCAAT enhancer binding protein beta
CHMP4B	charged multivesicular body protein 4B
CLCN3	chloride voltage-gated channel 3
COG3	component of oligomeric golgi complex 3
COPA	coatomer protein complex subunit alpha
COPB2	coatomer protein complex subunit beta 2
CREB3L2	cAMP responsive element binding protein 3 like 2
CSNK1A1	casein kinase 1 alpha 1
DDRGK1	DDRGK domain containing 1
DERL1	derlin 1
DERL2	derlin 2
DNAJB9	DnaJ heat shock protein family (Hsp40) member B9
DNAJC3	DnaJ heat shock protein family (Hsp40) member C3
DOP1B	DOP1 leucine zipper like protein B
DYSF	dysferlin

EDEM1	ER degradation enhancing alpha-mannosidase like protein 1
EDEM2	ER degradation enhancing alpha-mannosidase like protein 2
EDEM3	ER degradation enhancing alpha-mannosidase like protein 3
EHD3	EH domain containing 3
EIF2AK3	eukaryotic translation initiation factor 2 alpha kinase 3
EP300	E1A binding protein p300
EPB41L3	erythrocyte membrane protein band 4.1 like 3
ERLEC1	endoplasmic reticulum lectin 1
ERN1	endoplasmic reticulum to nucleus signaling 1
EXOC8	exocyst complex component 8
FAF2	Fas associated factor family member 2
FKBP14	FKBP prolyl isomerase 14
GABARAPL2	GABA type A receptor associated protein like 2
GBF1	golgi brefeldin A resistant guanine nucleotide exchange factor 1
GET4	golgi to ER traffic protein 4
GFPT1	glutamine--fructose-6-phosphate transaminase 1
GOLGA2	golgin A2
GOLGA4	golgin A4
GOLGA5	golgin A5
GOPC	golgi associated PDZ and coiled-coil motif containing
GORASP2	golgi reassembly stacking protein 2
GOSR2	golgi SNAP receptor complex member 2
HERPUD1	homocysteine inducible ER protein with ubiquitin like domain 1
HOOK3	hook microtubule tethering protein 3
HYOU1	hypoxia up-regulated 1
IER3IP1	immediate early response 3 interacting protein 1
INSIG1	insulin induced gene 1
ITPR1	inositol 1,4,5-trisphosphate receptor type 1
KDELR2	KDEL endoplasmic reticulum protein retention receptor 2
KIF13A	kinesin family member 13A
LEMD3	LEM domain containing 3
LMAN1	lectin, mannose binding 1
LMAN2	lectin, mannose binding 2
LMNA	lamin A/C
LNPK	lunapark, ER junction formation factor
LYST	lysosomal trafficking regulator
MCFD2	multiple coagulation factor deficiency 2
MON2	MON2 homolog, regulator of endosome-to-Golgi trafficking
NDEL1	nudE neurodevelopment protein 1 like 1

NPLOC4	NPL4 homolog, ubiquitin recognition factor
NRBF2	nuclear receptor binding factor 2
NSF	N-ethylmaleimide sensitive factor, vesicle fusing ATPase
NUP50	nucleoporin 50
NUP58	nucleoporin 58
P4HB	prolyl 4-hydroxylase subunit beta
PACSIN2	protein kinase C and casein kinase substrate in neurons 2
PDCD6IP	programmed cell death 6 interacting protein
PDIA3	protein disulfide isomerase family A member 3
PITPNB	phosphatidylinositol transfer protein beta
PMAIP1	phorbol-12-myristate-13-acetate-induced protein 1
PPP1R15B	protein phosphatase 1 regulatory subunit 15B
PREB	prolactin regulatory element binding
PRKCA	protein kinase C alpha
PTEN	phosphatase and tensin homolog
RAB14	RAB14, member RAS oncogene family
RAB1A	RAB1A, member RAS oncogene family
RAB22A	RAB22A, member RAS oncogene family
RAB2A	RAB2A, member RAS oncogene family
RAB31	RAB31, member RAS oncogene family
RABIF	RAB interacting factor
REEP1	receptor accessory protein 1
RER1	retention in endoplasmic reticulum sorting receptor 1
RNF103	ring finger protein 103
RNF139	ring finger protein 139
SAR1A	secretion associated Ras related GTPase 1A
SAR1B	secretion associated Ras related GTPase 1B
SDF2	stromal cell derived factor 2
SDF2L1	stromal cell derived factor 2 like 1
SEC13	SEC13 homolog, nuclear pore and COPII coat complex component
SEC16A	SEC16 homolog A, endoplasmic reticulum export factor
SEC22B	SEC22 homolog B, vesicle trafficking protein (gene/pseudogene)
SEC24A	SEC24 homolog A, COPII coat complex component
SEC24C	SEC24 homolog C, COPII coat complex component
SEC24D	SEC24 homolog D, COPII coat complex component
SEC31A	SEC31 homolog A, COPII coat complex component
SEC61A1	SEC61 translocon alpha 1 subunit
SEC62	SEC62 homolog, preprotein translocation factor
SEC63	SEC63 homolog, protein translocation regulator

SEL1L	SEL1L adaptor subunit of ERAD E3 ubiquitin ligase
SELENOS	selenoprotein S
SERP1	stress associated endoplasmic reticulum protein 1
SH3GLB1	SH3 domain containing GRB2 like, endophilin B1
SH3TC2	SH3 domain and tetratricopeptide repeats 2
SNX9	sorting nexin 9
SQSTM1	sequestosome 1
SRPRA	SRP receptor subunit alpha
SRPRB	SRP receptor subunit beta
SSR1	signal sequence receptor subunit 1
STAM2	signal transducing adaptor molecule 2
STT3B	STT3 oligosaccharyltransferase complex catalytic subunit B
STX5	syntaxin 5
SURF4	surfeit 4
SYNJ1	synaptojanin 1
SYVN1	synoviolin 1
TBC1D20	TBC1 domain family member 20
TBPL1	TATA-box binding protein like 1
TEX261	testis expressed 261
TFG	trafficking from ER to golgi regulator
TMCO1	transmembrane and coiled-coil domains 1
TMED10	transmembrane p24 trafficking protein 10
TMED2	transmembrane p24 trafficking protein 2
TMED5	transmembrane p24 trafficking protein 5
TMED9	transmembrane p24 trafficking protein 9
TMEM33	transmembrane protein 33
TMF1	TATA element modulatory factor 1
TMUB1	transmembrane and ubiquitin like domain containing 1
TOR1AIP2	torsin 1A interacting protein 2
TRIP11	thyroid hormone receptor interactor 11
TSPYL2	TSPY like 2
UFL1	UFM1 specific ligase 1
UFM1	ubiquitin fold modifier 1
UGGT1	UDP-glucose glycoprotein glucosyltransferase 1
USP25	ubiquitin specific peptidase 25
VAMP2	vesicle associated membrane protein 2
VCP	valosin containing protein
VMP1	vacuole membrane protein 1
VPS37A	VPS37A subunit of ESCRT-I

WASL	WASP like actin nucleation promoting factor
WIPI1	WD repeat domain, phosphoinositide interacting 1
XBP1	X-box binding protein 1
YIPF5	Yip1 domain family member 5

Supplementary Table 4. APA information about genes encoding highly expressed secreted proteins in SCTs[1]

Gene symbol	No. of PASs detected by 3'READS	Log2Ratio, SCT far cells vs. VCT far cells
CGA	1	4.2
CGB1	1	4.9
CGB2	1	5.4
CGB3	1	4.8
CGB5	1	4.5
CGB7	1	5.4
CGB8	1	4.5
CSH1	1	4.7
CSH2	1	6.4
CSHL1	1	7.8
GDF15	1	6.4
GH2	1	10.4
PSG1	1	11.1
PSG11	1	9.3
PSG3	1	11.2
PSG4	1	8.6
PSG6	1	11.4
PSG9	1	9.9

[1]Protein secretion property was based on the literature and Gene Ontology annotation.

Supplementary Table 5. Data sets used in this study

**Data set**	**Source**	**Data Identifier**
hESC to TB differentiation (3'READS)	This study	GEO: GSE138759
mESC to TB differentiation (3'READS)	This study	GEO: GSE138759
BeWo cell RNA stability (3'READS)	This study	GEO: GSE138759
hESC lineage (RNA-seq)	Xie et al., 2013	GEO: GSE16256
hESC-differentiation and cell size fractionation (RNA-seq)	Yabe et al., 2016	GEO: GSE73017
BeWo treatment with forskolin (RNA-seq)	Azar et al., 2018	SRA: SRP128121
Primary human trophoblast *ex vivo* differentiation (RNA-seq)	Yabe et al., 2016	GEO: GSE73017
Primary human trophoblast *ex vivo* differentiation (RNA-seq)	Azar et al., 2018	SRA: SRP128121
Placental single cell data (scRNA-seq)	Liu et al., 2018	GEO: GSE89497
Placental single cell data (scRNA-seq)	Tsang et al., 2017	EGA: EGAS00001002449
Placental single cell data (scRNA-seq)	Vento-Tormo et al., 2018	ArrayExpress: E-MTAB-6701
NCI-60 cancer cell lines (RNA-seq)	Reinhold et al., 2019	SRA: SRP133178
Mouse B cell and plasma cells (RNA-seq)	Shi et al., 2015	GEO: GSE60927
Mouse germinal center B cells (RNA-seq)	Ise et al., 2018	GEO: GSE109732
AtT-20 cell line overexpression (RNA-seq)	Khetchoumian et al., 2019	GEO: GSE132321
C2C12 differentiation (3'READS)	Wang et al. 2019	GEO: GSE115232
T cell activation (RNA-seq)	Pramanik et al., 2018	ArrayExpress: E-MTAB-6894

Supplementary Figure 1

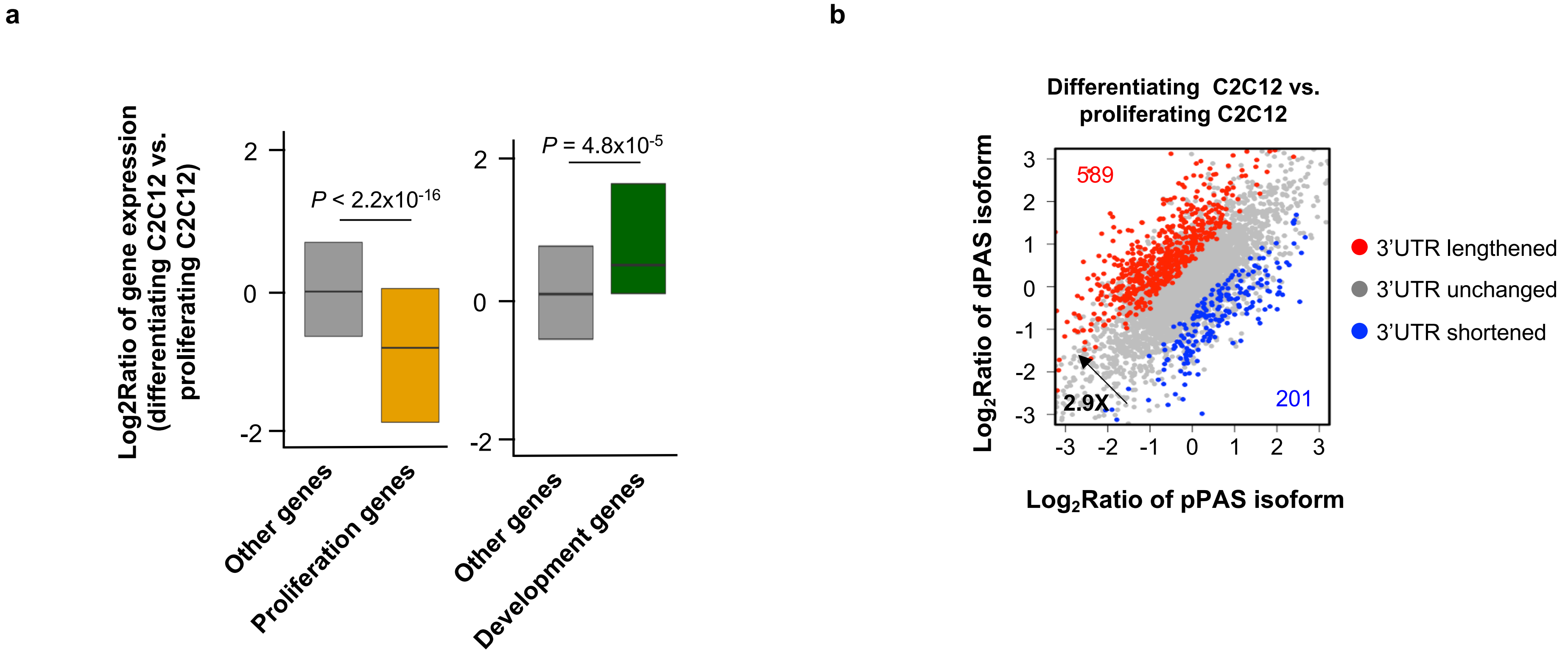

Supplementary Fig. 1. Regulation of proliferation genes and development genes in C2C12 differentiation. a. Box plots showing expression changes of cell proliferation genes (left, 340 genes) and development genes (right, 67 genes) in differentiating C2C12 cells vs. proliferating C2C12 cells. *P*-values (Wilcoxon test) indicating significance of difference between gene sets are shown. The data is based on Wang *et al*. (NCBI GEO: GSE115232) with two replicates. **b.** Scatter plot comparing 3'UTR APA isoform expression in differentiating C2C12 cells vs. proliferating C2C12 cells. Each dot is a gene with two selected 3'UTR APA isoforms. The number of genes with significantly lengthened 3'UTRs (red) or shortened 3'UTRs (blue) is indicated, and so is their ratio. Significance is based on $P < 0.05$ (DEXSeq) and >20% change of relative expression of two isoforms.

Supplementary Fig. 2

a

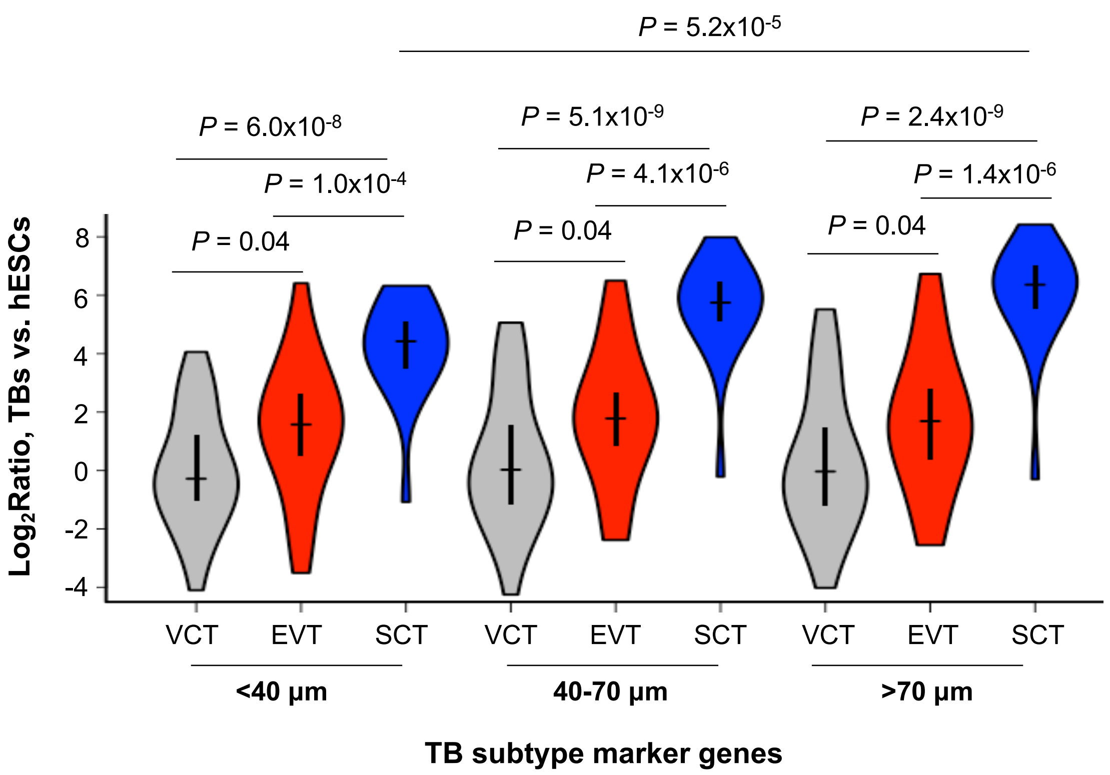

b

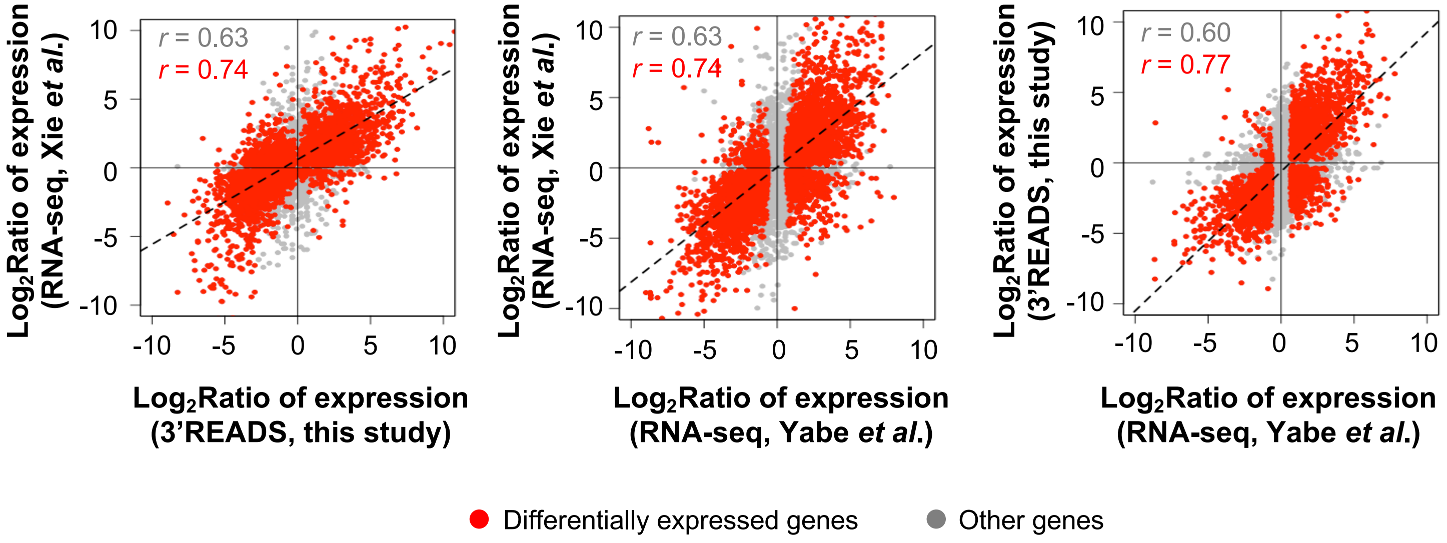

Supplementary Fig. 2. Additional data on hESC models. a. Violin plot showing gene expression changes of TB subtype marker genes in large TBs (>70 µm), medium TBs (40-70 µm), or small TBs (<40 µm) vs. hESCs. *P*-values (Wilcoxon test) for difference between gene sets are shown. **b.** Scatter plots comparing the three hESC-based TB models. Large TBs vs. hESCs is used for the Yabe *et al*. model. Pearson correlation coefficients based on all genes (grey) or differentially expressed genes (red, $P < 0.05$) are indicated.

Supplementary Fig. 3

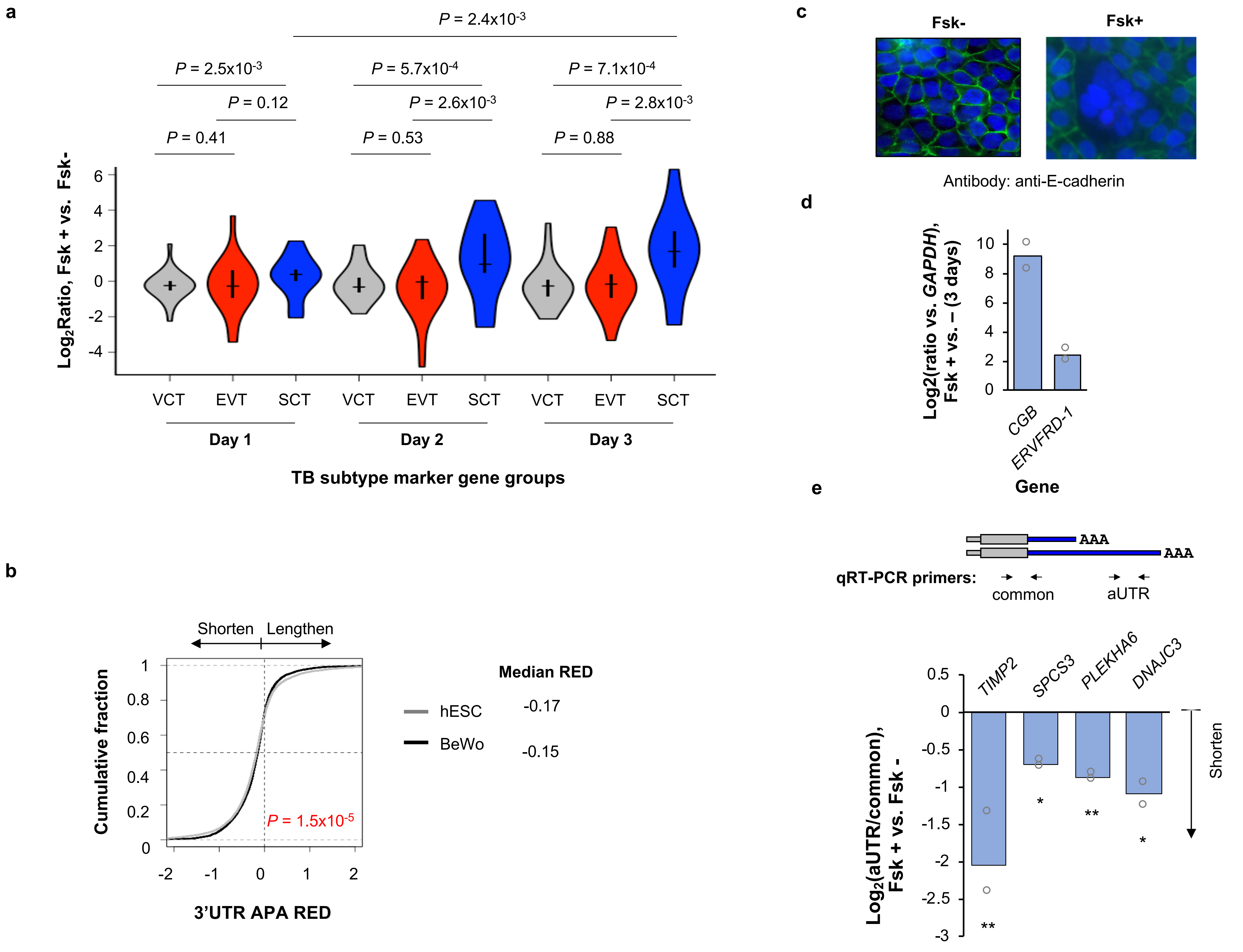

Supplementary Fig. 3. Additional data on syncytialization of BeWo cells. a. Violin plot showing gene expression changes of TB subtype marker genes in day 1, day 2, or day 3 samples (all Fsk+) vs. day 0 sample (Fsk-). *P*-values (Wilcoxon test) for difference between gene sets are shown. **b.** CDF curves comparing 3'UTR APA REDs in hESC and BeWo RNA-seq data for all APA genes (7,133 in total). Median RED and *P*-value (K-S test) for difference between data from the two models are indicated. **c.** Immunostaining of E-cadherin (green) in BeWo cells with or without Fsk treatment for 72 hr. Blue, DAPI stain. Representative image of two experiments. **d.** Gene expression changes of SCT marker genes *CGB* and *ERVFRD-1* by RT-qPCR in BeWo cells with or without Fsk treatment. Data are presented as mean ± standard deviation of two biological replicates. **e.** Top, schematic of primers designed for analysis of 3'UTR APA isoforms by RT-qPCR. Two primer sets are used for each gene, targeting a common region of both APA isoforms and the aUTR, respectively. Bottom, RT-qPCR analysis of 3'UTR APA between BeWo cells with vs. without Fsk treatment. Data are presented as mean ± standard deviation of two replicates. Significance of APA difference (t-test) between Fsk+ and Fsk- samples is indicated. **, $P < 0.01$; *, $P < 0.05$.

Supplementary Fig. 4

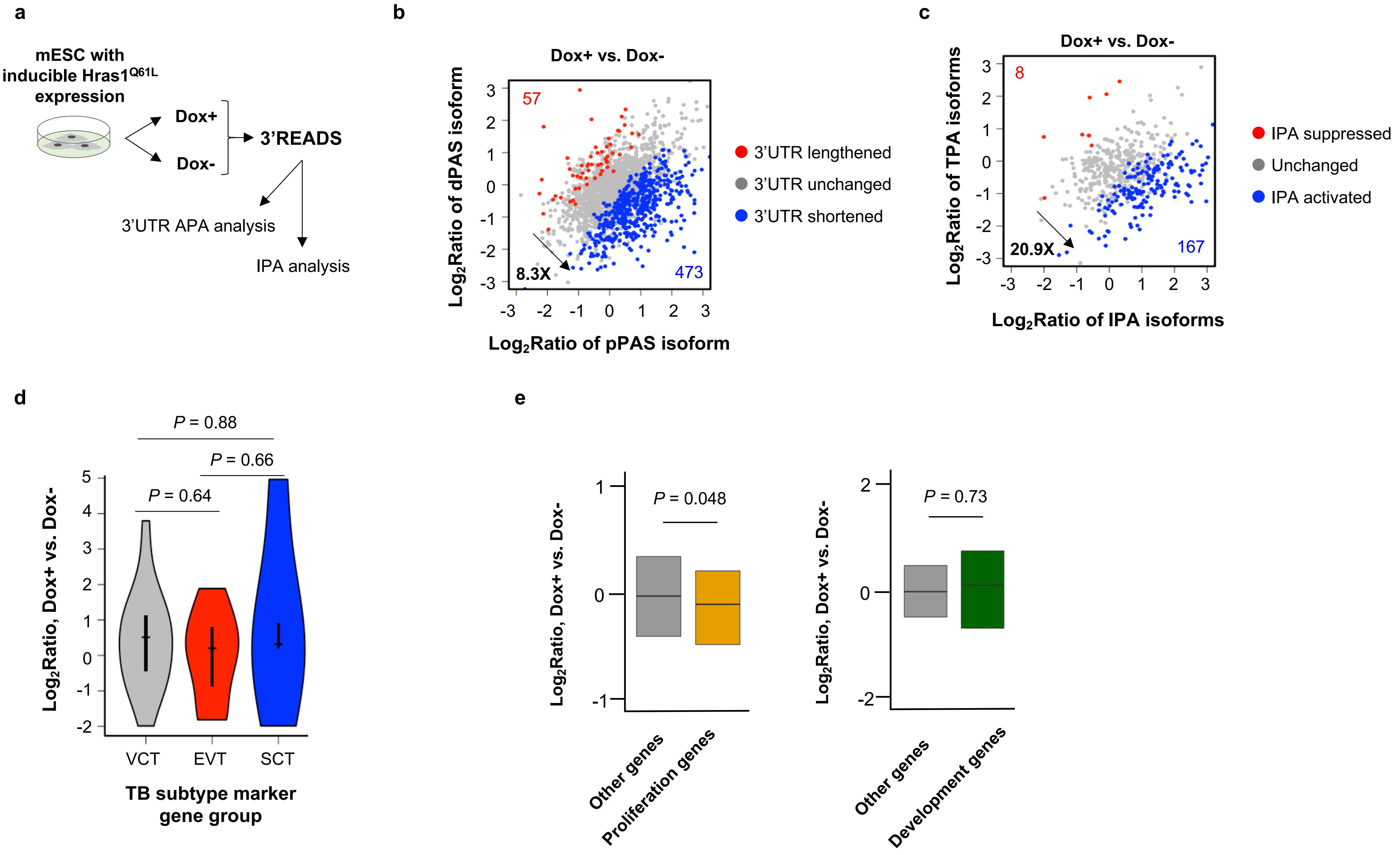

Supplementary Fig. 4. Mouse ESC model of TB differentiation. a. Schematic of experimental design. Mouse embryonic stem cells (mESCs) are differentiated into syncytial giant cells by ectopic expression of *Hras1* (Q61L) after doxycycline (Dox) induction. RNA samples are subject to 3'READS analysis. **b.** Scatter plot showing expression difference of 3'UTR APA isoforms in Dox+ vs. Dox- samples. Each dot is a gene with two selected 3'UTR APA isoforms. The number of genes with significantly lengthened 3'UTRs (red) or shortened 3'UTRs (blue) is indicated, and so is their ratio. Significance is based on $P < 0.05$ (DEXSeq) and >20% change of relative expression of the two isoforms. **c.** As in b., except that IPA isoforms vs. TPA isoforms is shown. **d.** Violin plot showing TB subtype marker gene expression changes in Dox+ vs. Dox- cells. *P*-value (Wilcoxon test) indicating significance of difference is shown. **e.** Box plots showing gene expression changes of cell proliferation genes (left, 262 genes) or development genes (right, 67 genes) compared to other genes in Dox+ vs. Dox- cells. *P*-value (Wilcoxon test) indicating significance of difference is shown.

Supplementary Fig. 5

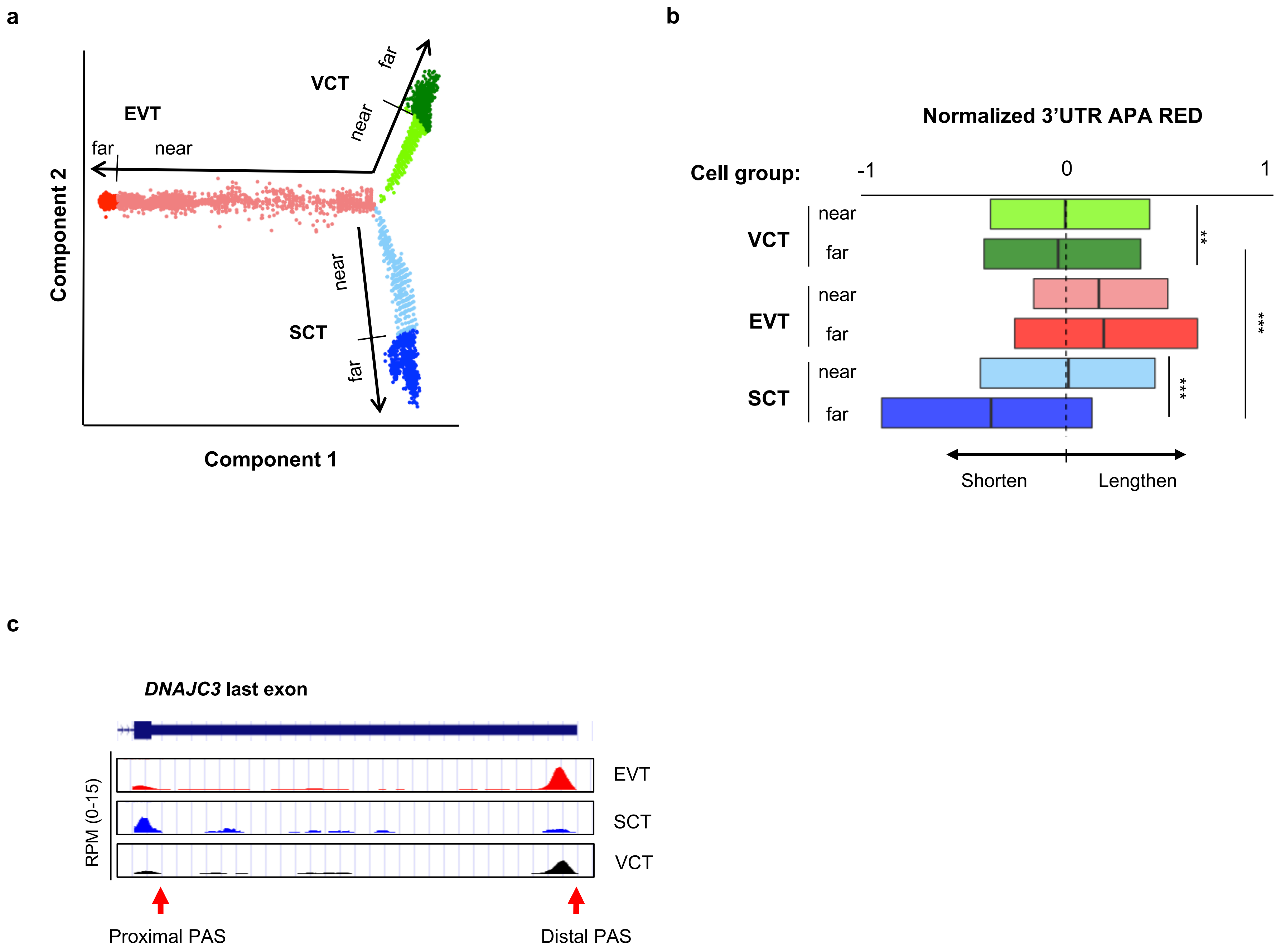

Supplementary Fig. 5. Single cell analysis of third trimester placental samples. a. Pseudotime analysis of TB cells (four normal term pregnancies in the Tsang *et al.* study). The trajectories of TB cells, plotted by DDRTree, are based on expression of TB subtype marker genes. Each lineage is divided into two halves, far and near. Cells in each group are colored as indicated. **b.** 3'UTR APA REDs (3,893 genes) for different TB types and subgroups (corresponding to those in penel a.). 3'UTR APA REDs are normalized to the mean of all groups. Significance of APA difference (Wilcoxon test) between groups is indicated. **c.** UCSC Genome Browser tracks showing single cell data from the Tsang *et al.* study for *DNAJC3.*

Supplementary Fig. 6

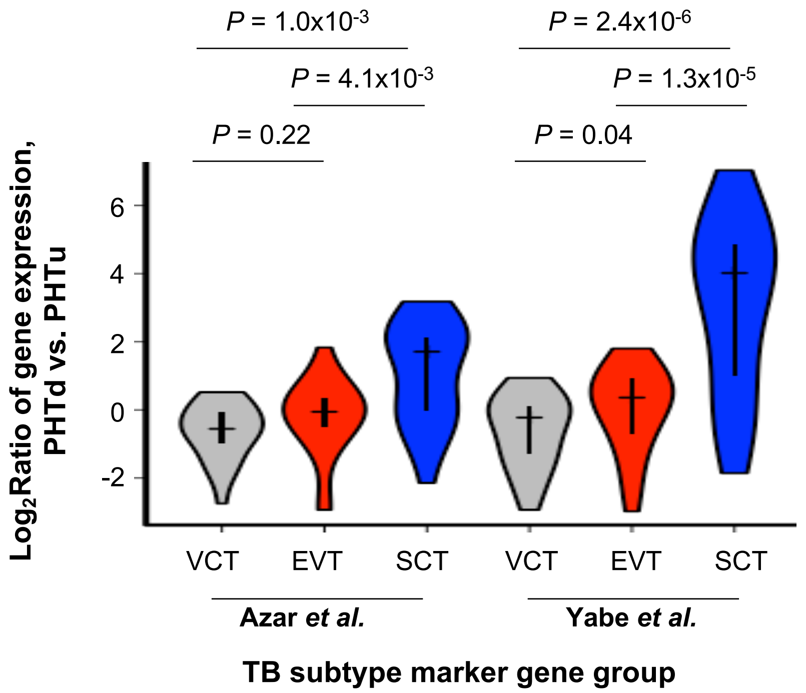

Supplementary Fig. 6. TB subtype marker gene expression in *in vitro* syncytialization of primary human trophoblast (PHT) cells. Violin plot showing gene expression changes of TB subtype marker genes in syncytialized PHTd cells vs. non-syncytialized PHTu cells. *P*-values (Wilcoxon test) for difference between gene sets are shown.

Supplementary Fig. 7

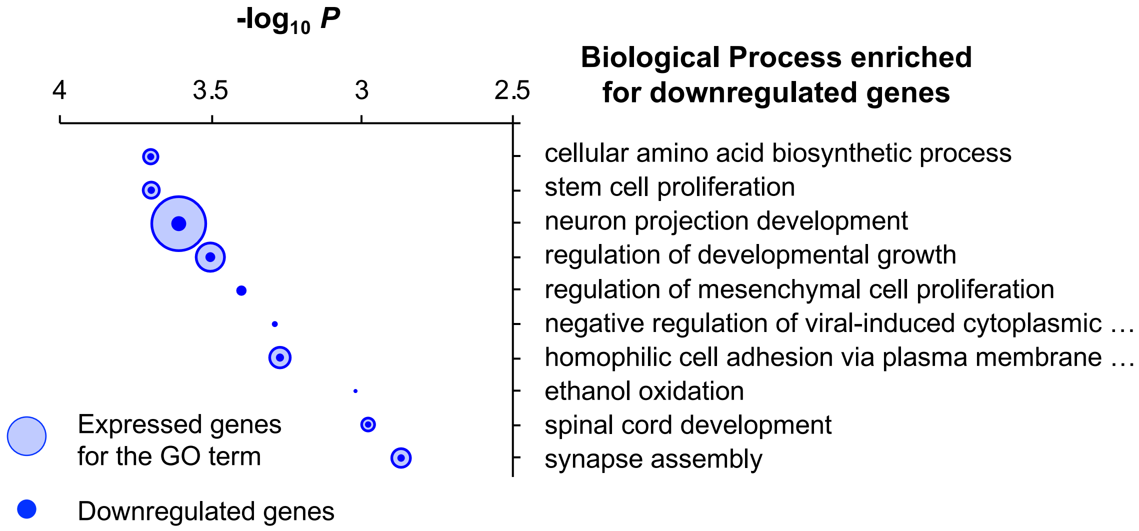

Supplementary Fig. 7. GO terms enriched for commonly downregulated genes in hESC and BeWo models. Data are presented as in Fig. 5c.

Supplementary Fig. 8

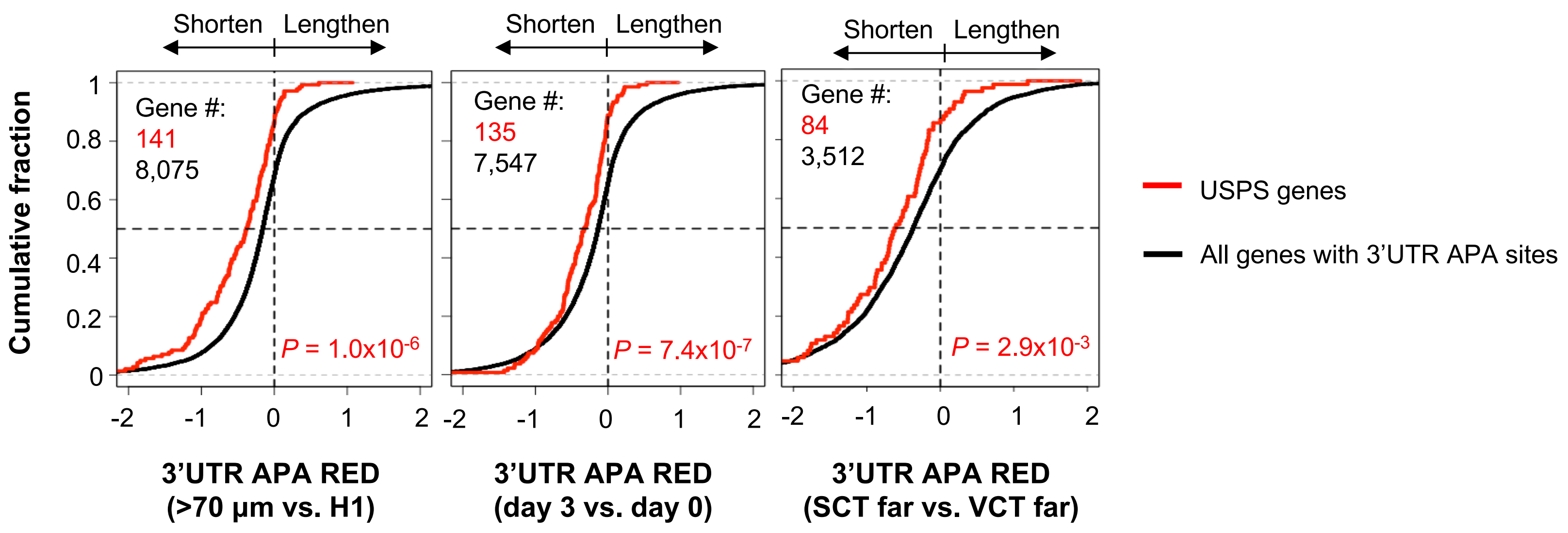

Supplementary Fig. 8. 3'UTR APA of USPS genes. CDF curves comparing 3'UTR APA REDs of USPS genes (red line) and all genes with 3'UTR APA sites in three datasets, as indicated. Number of genes in each plot is shown. *P*-value (K-S test) for difference between the two gene sets is indicated.

Supplementary Fig. 9

a

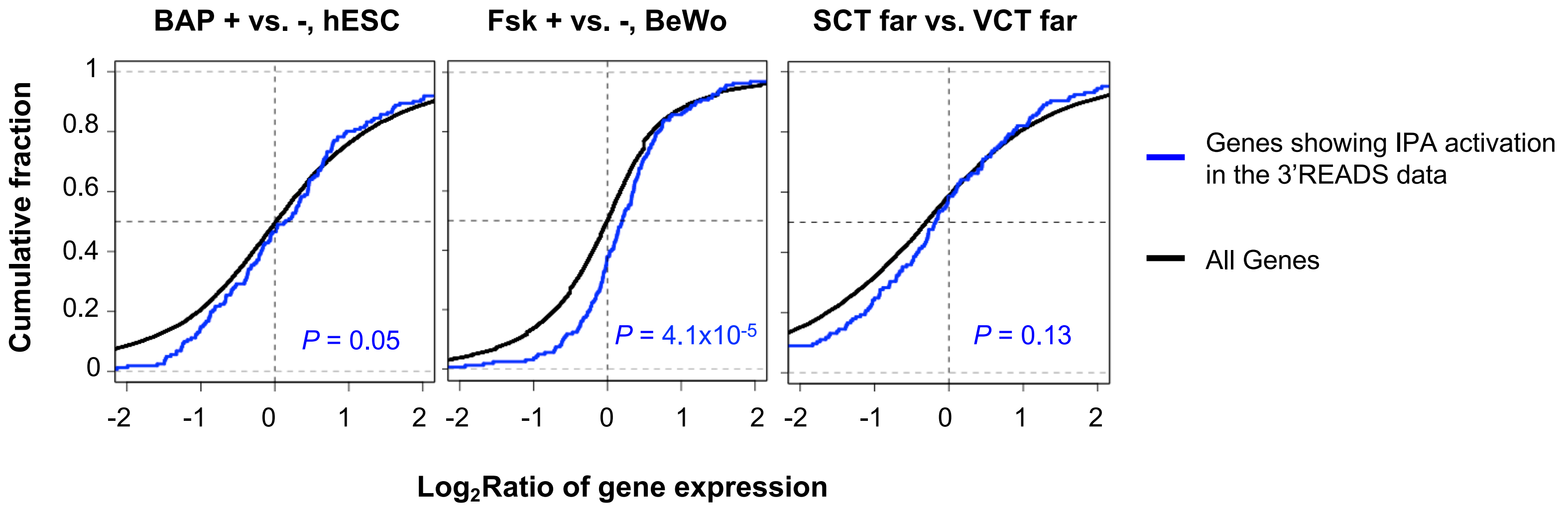

b

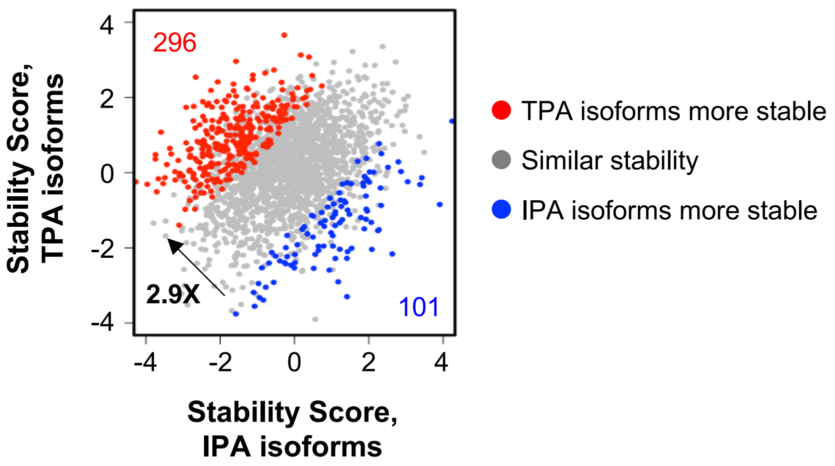

Supplementary Fig. 9. Gene expression changes vs. IPA regulation. a. Expression changes of genes with IPA activation (blue dots in Fig. 2f) in three datasets, as indicated. *P*-values are based on K-S test. **b.** Scatter plot comparing Stability Scores between IPA isoforms and 3' terminal exon (TPA) isoforms. Each dot represents a gene, for which all its IPA isoforms were combined and all its TPA isoforms are combined. Stability Scores for each group are averaged.

Supplementary Fig. 10

a

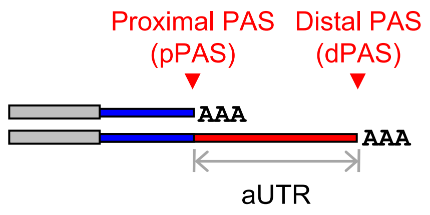

Enrichment in aUTRs of shortened genes

4-mer	$-\log_{10} P$
UUUU	21.2
UUGU	16.5
UUUG	15.6
GUUU	13.8
UUAA	9.8
GUGU	9.2
UGUU	8.8
UGUG	8.6
GGGG	8.1
UUUA	7.9

b

Enriched for 3'UTRs of unstable transcripts (bottom 20% based on SS)

4-mer	$-\log_{10} P$
UUUU	Inf
UUUA	202.8
AUUU	195.3
UAUU	184.1
UUAA	171.3
UUAU	151.2
AUAU	145.3
UAAA	141.7
UAUA	139.2
AAAU	137.8

Enriched for 3'UTRs of stable transcripts (top 20% based on SS)

4-mer	$-\log_{10} P$
CCCC	Inf
GCCC	258.5
GGCC	212.0
CCCU	198.9
CCUG	191.5
GGGC	181.7
CCCA	180.9
CCUC	162.9
CUCC	153.3
UCCC	148.9

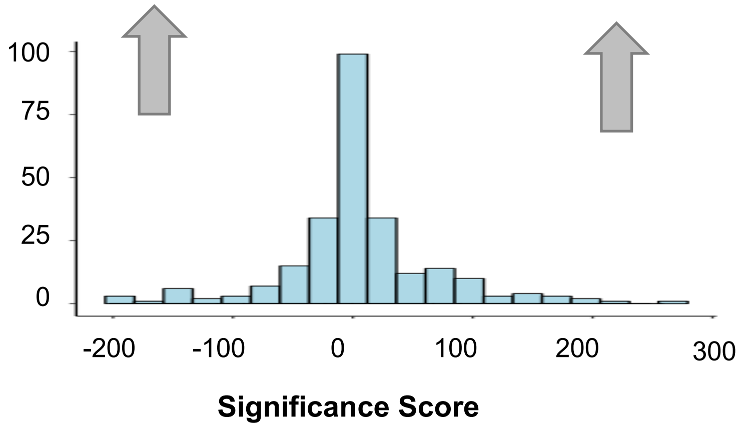

Supplementary Fig. 10. 3'UTR motif analysis. a. Tetramers enriched in the aUTRs of genes with 3'UTR shortening in differentiation of hESCs to TBs (blue dots in Fig. 2c). Top ten enriched tetramers are shown, and their *P*-values (Fisher's exact test) are indicated. **b.** Tetramers enriched in 3'UTRs of unstable transcripts (upper left) or stable transcripts (upper right). Top ten enriched tetramers in each group are shown, and their *P*-values (Fisher's exact test) are indicated. Distribution of tetramer significance scores is shown at the bottom. Significance score is $-\log_{10}(P)$*S, where *P* is based on the Fisher's exact test and S is 1 when enriched for stable transcripts or -1 when enriched for unstable transcripts.

Supplementary Fig. 11

a

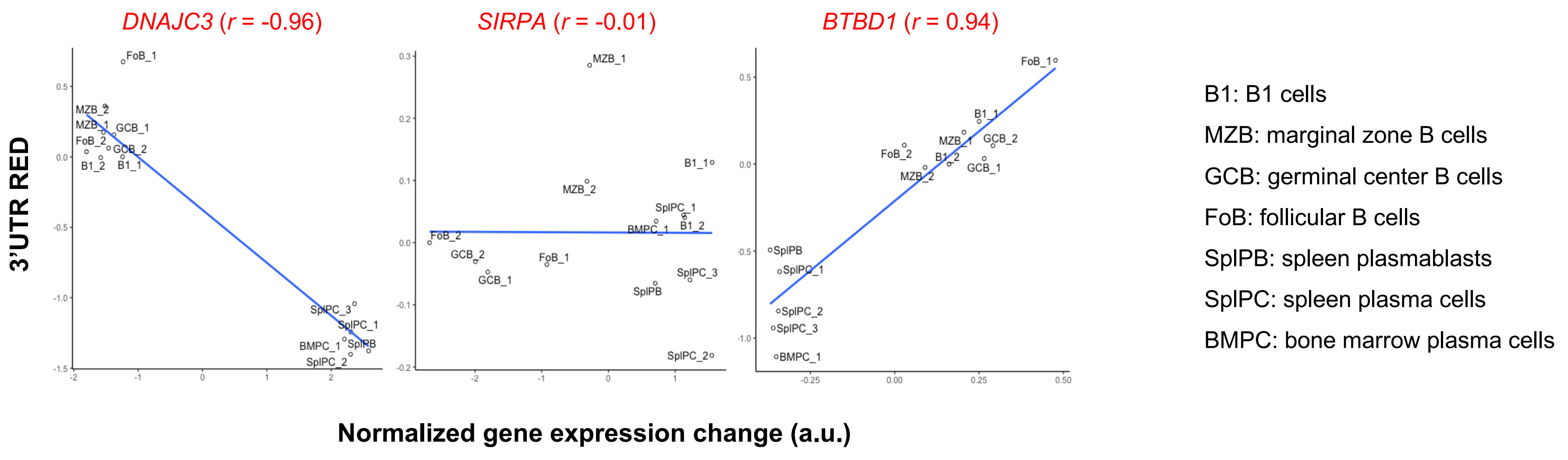

b

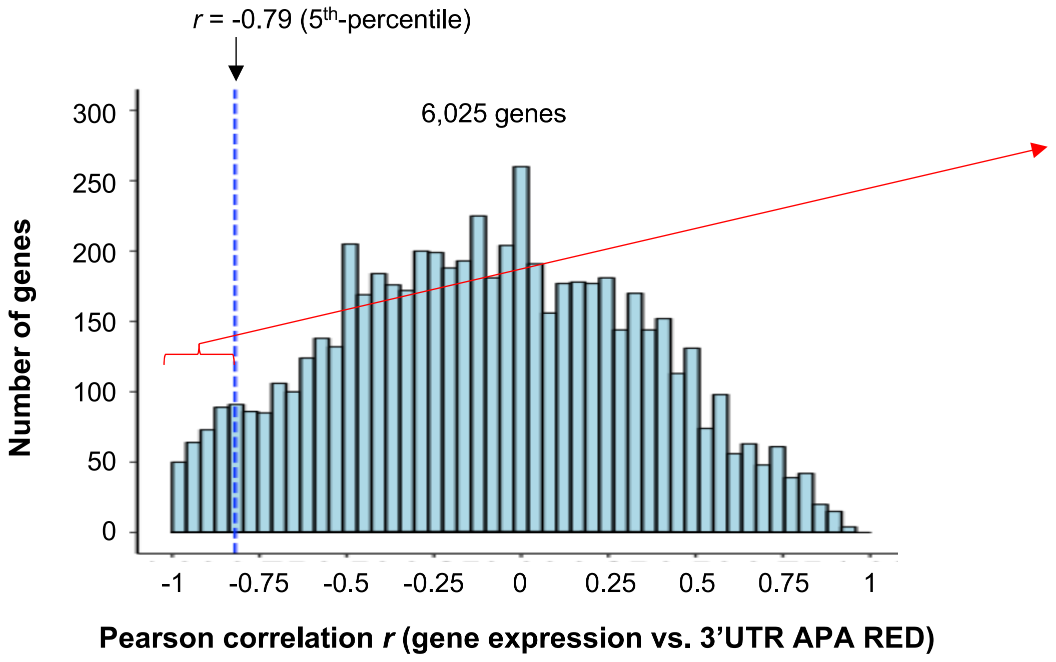

c

GO terms enriched for genes with *r* < -0.79

P-value	GO term (Biological Process)
1.6E-05	negative regulation of response to endoplasmic reticulum stress
4.1E-05	protein exit from endoplasmic reticulum
2.1E-03	biological phase
2.2E-03	endoplasmic reticulum unfolded protein response
2.9E-03	cellular response to interleukin-4
5.6E-03	intracellular protein transmembrane transport
6.8E-03	cellular response to biotic stimulus
7.0E-03	protein carboxylation
7.0E-03	regulation of phospholipase A2 activity
7.0E-03	skin morphogenesis

Supplementary Fig. 11. Analysis of B cell and plasma cell data. a. Scatter plots showing correlation between 3'UTR APA REDs and gene expression levels across B cells and plasma cells. Three example genes are shown, representing positively correlated (left), negatively correlated (right), and not correlated (middle) cases. Gene name and Pearson correlation coefficient *r* are shown in each plot. **b.** Distribution of Pearson correlation coefficient *r* for 6,025 genes. The bottom 5th-percentile is indicated. **c.** GO terms enriched for genes in the bottom 5% of the distribution (from panel b).

Supplementary Fig. 12

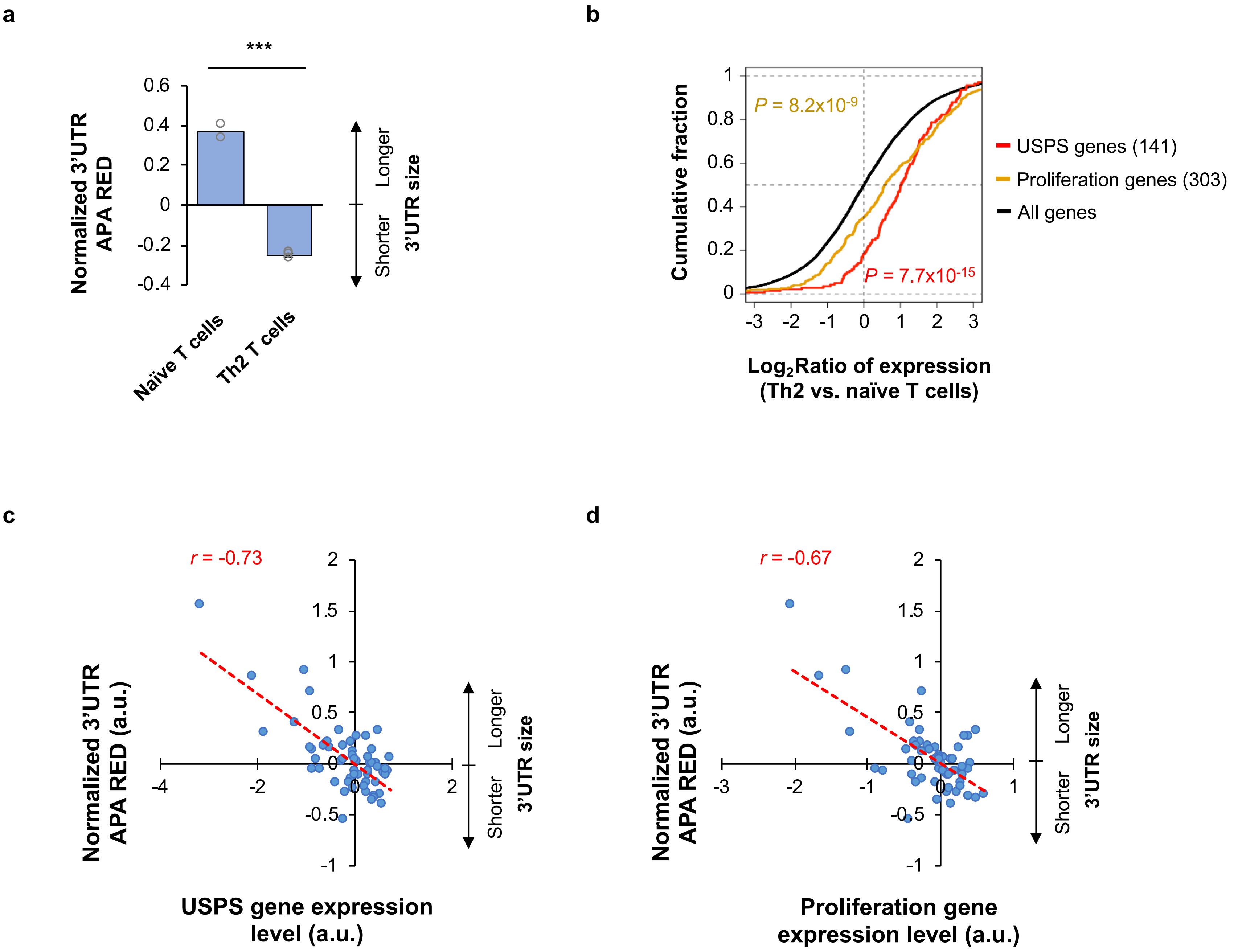

Supplementary Fig. 12. Evidence that secretion and proliferation genes are coupled in some systems. a. 3'UTR APA REDs for activated Th2 cells (3 replicates) vs. naïve T cells (2 replicates), based on a study by Pramanik *et al*. Error bars are standard deviation of two (naïve T cells) or three (Th2 cells) replicates. Significance of difference (t-test) between cell groups is indicated. 3'UTR APA REDs were normalized to median of all samples. **b.** CDF curves of gene expression changes for USPS genes (red) or proliferation genes (orange) in Th2 cells vs. naïve T cells. *P*-values (K-S test) for differences between gene sets are indicated. **c & d.** Scatter plots showing correlation between 3'UTR REDs and USPS gene (c) or proliferation gene (d) expression levels across NCI-60 cancer cell lines (a study by Reinhold *et al*.). 3'UTR APA REDs are normalized to the median of all samples. Pearson correlation coefficient (*r*) is indicated.
