# Supplementary material for: Widespread transcript shortening through alternative polyadenylation in secretory cell differentiation
Source: Nat Commun. 2020 Jun 23;11:3182. doi: 10.1038/s41467-020-16959-2 (PMC7311474; doi:10.1038/s41467-020-16959-2)
Supplement: Supplementary file 3 — Reporting Summary [file 41467_2020_16959_MOESM3_ESM.pdf]

nature research

Corresponding author(s): Bin Tian

Last updated by author(s): May 15, 2020

Reporting Summary

Nature Research wishes to improve the reproducibility of the work that we publish. This form provides structure for consistency and transparency in reporting. For further information on Nature Research policies, see Authors & Referees and the Editorial Policy Checklist.

Statistics

For all statistical analyses, confirm that the following items are present in the figure legend, table legend, main text, or Methods section.

n/a	Confirmed	
☐	☒	The exact sample size (*n*) for each experimental group/condition, given as a discrete number and unit of measurement
☐	☒	A statement on whether measurements were taken from distinct samples or whether the same sample was measured repeatedly
☐	☒	The statistical test(s) used AND whether they are one- or two-sided<br>*Only common tests should be described solely by name; describe more complex techniques in the Methods section.*
☒	☐	A description of all covariates tested
☒	☐	A description of any assumptions or corrections, such as tests of normality and adjustment for multiple comparisons
☐	☒	A full description of the statistical parameters including central tendency (e.g. means) or other basic estimates (e.g. regression coefficient) AND variation (e.g. standard deviation) or associated estimates of uncertainty (e.g. confidence intervals)
☐	☒	For null hypothesis testing, the test statistic (e.g. *F*, *t*, *r*) with confidence intervals, effect sizes, degrees of freedom and *P* value noted<br>*Give P values as exact values whenever suitable.*
☒	☐	For Bayesian analysis, information on the choice of priors and Markov chain Monte Carlo settings
☒	☐	For hierarchical and complex designs, identification of the appropriate level for tests and full reporting of outcomes
☐	☒	Estimates of effect sizes (e.g. Cohen's *d*, Pearson's *r*), indicating how they were calculated

Our web collection on statistics for biologists contains articles on many of the points above.

Software and code

Policy information about availability of computer code

Data collection	Custom scripts for processing and analyzing data were written in R or Perl and are available upon request. Software packages used include CutAdapt (v1.17), Bowtie2 (v2.2.9), GenomicAlignments (v1.12.2), GenomicFeatures (v1.28.5), DESeq (v1.24.0), DEXSeq (v1.36.0), STAR (v2.5.2), GOstats (v2.44.0), CellRanger (v3.0.1), Samtools (v3.1.0), Seurat (v3.1.0), and monocle (v2.6.4).
Data analysis	Custom scripts for processing and analyzing data were written in R or Perl and are available upon request. Software packages used include CutAdapt (v1.17), Bowtie2 (v2.2.9), GenomicAlignments (v1.12.2), GenomicFeatures (v1.28.5), DESeq (v1.24.0), DEXSeq (v1.36.0), STAR (v2.5.2), GOstats (v2.44.0), CellRanger (v3.0.1), Samtools (v3.1.0), Seurat (v3.1.0), and monocle (v2.6.4).

For manuscripts utilizing custom algorithms or software that are central to the research but not yet described in published literature, software must be made available to editors/reviewers. We strongly encourage code deposition in a community repository (e.g. GitHub). See the Nature Research guidelines for submitting code & software for further information.

Data

Policy information about availability of data

All manuscripts must include a data availability statement. This statement should provide the following information, where applicable:

- Accession codes, unique identifiers, or web links for publicly available datasets
- A list of figures that have associated raw data
- A description of any restrictions on data availability

Sequencing data generated in this study have been deposited into the NCBI GEO database under the accession number GSE138759. All data sets used in this study are listed in Supplementary Table 5.

Field-specific reporting

Please select the one below that is the best fit for your research. If you are not sure, read the appropriate sections before making your selection.

- [x] Life sciences
- [ ] Behavioural & social sciences
- [ ] Ecological, evolutionary & environmental sciences

For a reference copy of the document with all sections, see nature.com/documents/nr-reporting-summary-flat.pdf

Life sciences study design

All studies must disclose on these points even when the disclosure is negative.

Sample size	Various sample sizes in different experiments as indicated in respective figure legends and Methods.
Data exclusions	None.
Replication	Experiments were repeated as indicated in respective figure legends and Methods.
Randomization	Cell line batches were random.
Blinding	Blinding is not applicable because all experiments were carried out with cell lines.

Reporting for specific materials, systems and methods

We require information from authors about some types of materials, experimental systems and methods used in many studies. Here, indicate whether each material, system or method listed is relevant to your study. If you are not sure if a list item applies to your research, read the appropriate section before selecting a response.

Materials & experimental systems

n/a	Involved in the study	
☐	☒	Antibodies
☐	☒	Eukaryotic cell lines
☒	☐	Palaeontology
☒	☐	Animals and other organisms
☒	☐	Human research participants
☒	☐	Clinical data

Methods

n/a	Involved in the study	
☒	☐	ChIP-seq
☒	☐	Flow cytometry
☒	☐	MRI-based neuroimaging

Antibodies

Antibodies used	Rabbit anti-E-cadherin antibody: Cell Signaling, #3195.<br>FITC-conjugated goat anti-rabbit antibody: Jackson ImmunoResearch, 111-095-144.
Validation	These antibodies have been validated by the manufacturers and have been widely used in previous studies of other labs.

Eukaryotic cell lines

Policy information about cell lines

Cell line source(s)	Human embryonic stem cells (H9): Lab of James Manley (Columbia University)<br>BeWo cells: Lab of Sergei Kotenko (Rutgers New Jersey Medical School)<br>Mouse embryonic stem cells: Lab of Chi-Wei Lu (Rutgers Robert Wood Johnson Medical School)
Authentication	Cells are authenticated based on microscopic features and gene expression profiles.
Mycoplasma contamination	Cell lines are mycoplasma free.
Commonly misidentified lines<br>(See ICLAC register)	none
